# Cytokines in First-Episode Psychosis: Implications for Pathophysiology: A Narrative Literature Review

**DOI:** 10.3390/brainsci16080867

**Published:** 2026-08-16

**Authors:** Lindokuhle Thela, Bongani Nkambule, Zama Msibi, Vuyokazi Ntlantsana, Saeeda Paruk, Andrew Tomita, Khethelo Richman Xulu, Bonginkosi Chiliza

**Affiliations:** 1 Discipline of Human Physiology, School of Medicine, University of KwaZulu-Natal, 719 Umbilo Road, Durban 4000, South Africantlantsanav@ukzn.ac.za (V.N.); chilizab@ukzn.ac.za (B.C.); 2 Centre for Rural Health, School of Nursing and Public Health, University of KwaZulu-Natal, Durban 4001, South Africa; 3 School of Molecular and Cell Biology, The University of the Witwatersrand, Johannesburg 2050, South Africa

**Keywords:** psychosis, inflammation, cytokines, first-episode psychosis

## Abstract

**Highlights:**

**What are the main findings?**

- Immune system dysfunction is linked to first-episode psychosis (FEP).
- Environmental factors like maternal infection and childhood trauma may increase psychosis risk by altering immune responses.
- Inflammatory markers are often elevated in FEP, but results are inconsistent.
- Immune changes may drive certain symptoms and differences in psychosis.

**What are the implications of the main findings?**

- Identifying immune-related subgroups could improve personalized treatment.
- More research is needed before immune-based therapies can be widely used.
- Standardized studies are necessary to clarify immunity’s role in psychosis.

**Abstract:**

A pro-inflammatory state, characterized by elevated levels of pro-inflammatory cytokines, is frequently reported among individuals presenting with primary first-episode psychosis (FEP), particularly those with environmental risk factors such as maternal infections and early childhood traumatic experiences. These findings suggest that immune system disturbances may play a crucial role in the onset of psychotic disorders. Building on this, cytokines may contribute to the risk of FEP from the stage of neurodevelopment, where they can induce aberrant changes in neuronal growth. During early childhood, these cytokine-mediated processes may disrupt normal neuronal maturation and lead to brain alterations that increase vulnerability to primary psychotic disorders. Furthermore, pro-inflammatory cytokines exhibit strong bidirectional modulatory interactions with dopamine, a key neurotransmitter implicated in the pathogenesis and persistence of psychosis. Notably, these cytokines may also influence the clinical presentation and severity of psychosis. In addition, antipsychotic medications can partially modulate cytokine levels, and this modulation has been correlated with the efficacy of antipsychotics in treating various psychotic symptoms. Frequently reported cytokines in this context include interleukins (IL-1β, IL-2, IL-4, IL-6, IL-8, and IL-10), tumour necrosis factor-α (TNF-α), and interferon-gamma (IFN-γ). We conducted a non-systematized literature search on Google Scholar and PubMed for studies looking at cytokines in primary psychotic disorders during FEP. In this narrative review, we provide an overview of the literature on immune dysregulation in FEP, with a particular emphasis on cytokines.

## 1. Introduction

Schizophrenia and related psychotic disorders affect approximately 1% of the global population and constitute a major source of disability among young adults, with first-episode psychosis (FEP) typically presenting in adolescence or early adulthood [1,2,3]. Schizophrenia, the most prevalent primary psychotic disorder, is marked by a severe, chronic clinical trajectory and is associated with a reduction in life expectancy of up to 17 years [4]. Despite its relatively low prevalence, schizophrenia exerts a substantial impact on global health, contributing disproportionately to disability-adjusted life years worldwide [3]. Schizophrenia is clinically heterogeneous, with outcomes shaped by onset age, untreated duration, comorbidities, and social factors [5,6,7,8]. Negative and cognitive symptoms are often treatment-resistant [9,10]. While traditional models have emphasized disruptions in dopamine and glutamate neurotransmission to explain the hallmark cognitive, emotional, and perceptual disturbances of psychosis, converging evidence now implicates immune system dysregulation, particularly involving cytokines, as a key factor in disease pathophysiology [11]. Converging evidence indicates elevated pro-inflammatory cytokine levels in FEP, underscoring the importance of understanding these cytokines during this period [11,12]. These immune changes interact with neurotransmitter systems, influencing clinical presentation and disease trajectory. Chronic antipsychotic exposure further alters immune profiles and induces metabolic disturbances, complicating immunological assessment [13]. This narrative literature review synthesizes current research on cytokine alterations in FEP and explores their implications for our understanding of psychosis pathophysiology, with the aim of informing future therapeutic strategies.

## 2. Methods and Literature Search

**Figure 1:**
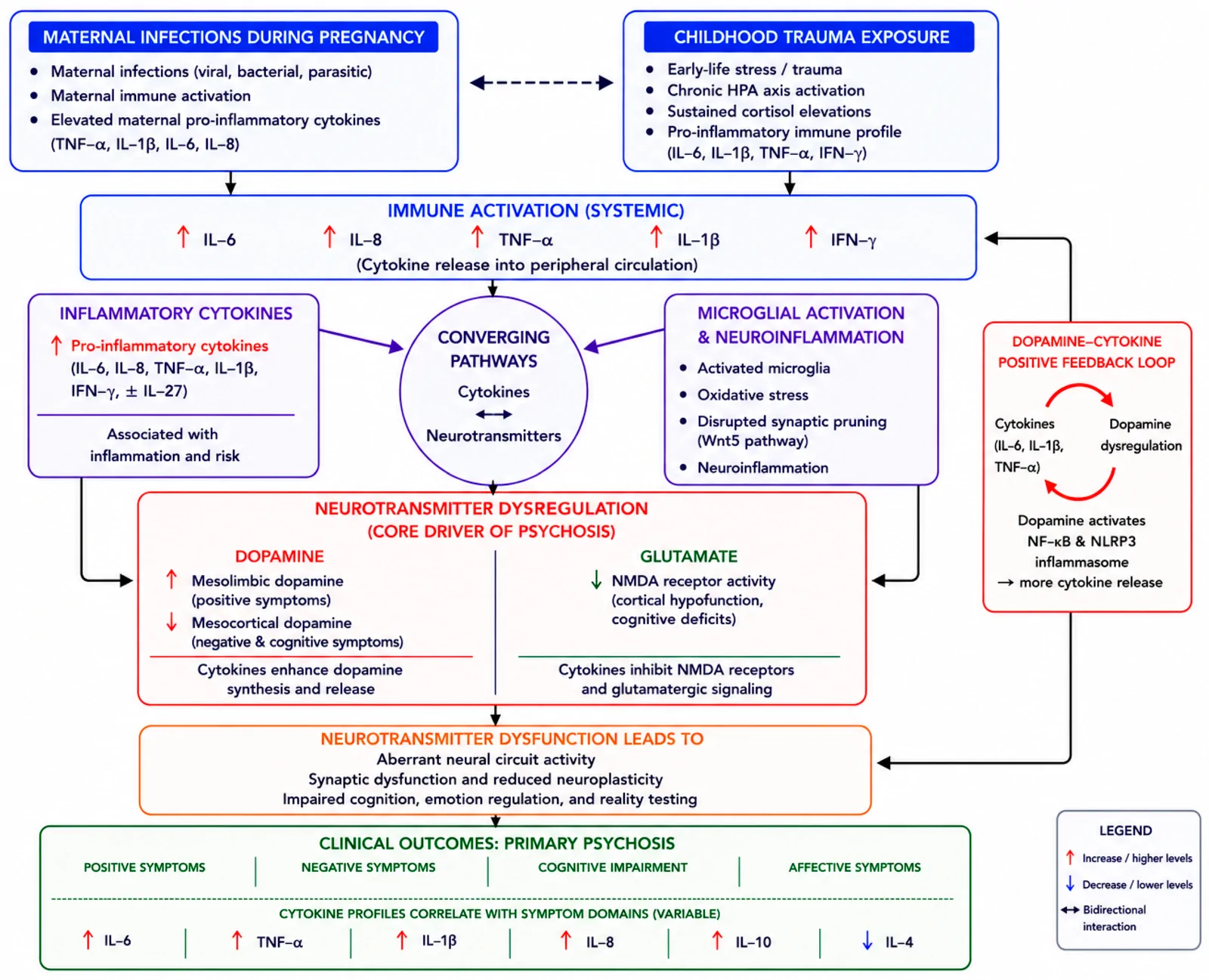
Simplified schematic overview of immune dysregulation in primary psychosis. Early-life risk factors, such as maternal infection and childhood trauma, trigger systemic immune activation and elevated pro-inflammatory cytokines (e.g., IL-6, IL-8, TNF-α, IL-1β, IFN-γ). These cytokines interact with microglia, drive neuroinflammation, and disrupt dopaminergic and glutamatergic pathways. Elevated cytokines increase mesolimbic dopamine levels, contribute to mesocortical hypodopaminergia, and inhibit NMDA receptor-mediated glutamatergic signalling, leading to synaptic dysfunction and impaired neuroplasticity. A positive feedback loop between cytokines and dopamine amplifies neuroinflammation by activating the NF-κB and NLRP3 inflammasome pathways. These mechanisms underlie the core symptoms of primary psychosis, although associations between specific cytokines and symptoms remain variable across studies. Abbreviations: HPA, hypothalamic–pituitary–adrenal; IFN-γ, interferon-gamma; IL, interleukin; NF-κB, nuclear factor kappa B; NLRP3, nucleotide-binding oligomerization domain-like receptor family pyrin domain-containing 3 inflammasome; NMDA, N-methyl-D-aspartate; TNF-α, tumour necrosis factor-alpha.

This narrative review explored the relationship between psychosis, including the first episode of primary psychotic disorder (schizophrenia spectrum disorders) and inflammatory mediators, with a focus on cytokines. The literature search, conducted via Google Scholar and PubMed, included studies reporting on cytokine alterations in primary FEP. Studies reporting on secondary causes of psychosis (e.g., autoimmune encephalitis) and other psychiatric disorders were excluded. Figure 1 was created using AI that was guided by the author. As this review does not follow PRISMA guidelines, detailed search terms are not included.

### Historical Background of Inflammation and Its Role in Primary Psychosis

The association between immune dysregulation and psychosis is increasingly recognized as a pivotal theme in neuropsychiatric research. Epidemiological investigations, including large-scale longitudinal cohorts and case-control studies, have shown that maternal infections during pregnancy are associated with an elevated risk of psychosis in offspring [14]. These observations have substantially shifted the understanding of psychosis from a disorder explained solely by neurotransmitter abnormalities to one in which early-life immune perturbations contribute to altered neurodevelopment and lifelong vulnerability. Among the infectious agents most frequently implicated are *Toxoplasma gondii* (*T. gondii*) and herpes simplex virus 2 [15,16]. *T. gondii* has particularly received attention because of its neurotrophic properties. The parasite can cross the placental barrier and infect the developing foetal brain, resulting in marked premature neuroinflammation during the critical period of neurodevelopment [17]. Experimental studies suggest that *T. gondii* can disrupt dopaminergic neurotransmission, in part by producing a variant of tyrosine hydroxylase, a rate-limiting enzyme in dopamine synthesis, thereby increasing dopamine production in infected neurons [17]. Given the longstanding role of dopamine dysregulation in the pathophysiology of psychosis, these findings provide a plausible biological link between prenatal infection and later psychotic illness.

While direct foetal infection may contribute to neuropathology in some cases, accumulating evidence suggests that maternal immune activation represents the principal mechanism linking prenatal infection to psychosis [18]. Rather than the pathogen itself causing widespread foetal injury, the maternal immune response generated during infection appears to exert more profound effects on the developing brain. Maternal infections are associated with increased production of cytokines and chemokines, including IL-6, IL-8, TNF-α, and other inflammatory mediators, thereby altering the intrauterine environment [18,19]. Exposure of the foetal brain to abnormal cytokine milieu during critical neurodevelopmental processes interfere with neuronal migration, proliferation, differentiation, synaptogenesis, and myelination, thereby increasing susceptibility to neurodevelopmental disorders, including psychotic disorders, schizophrenia, and other psychotic disorders [19].

Under physiological conditions, the placenta functions as a highly specialized immunological barrier that protects the developing foetus while simultaneously allowing tightly regulated maternal–foetal communication. Decidual cells and resident macrophages provide immune surveillance, offering significant protection to the developing embryo against pathogenic threats [20]. However, when maternal immune activation is pronounced, these defences may be overwhelmed, leading to foetal cytokine exposure and subsequent disruption of foetal brain development.

## 3. Role of Cytokines in Neurodevelopment

The neurodevelopmental hypothesis of schizophrenia increasingly proposes that disturbances occurring during early brain development predispose individuals to psychosis many years before clinical symptoms emerge [21]. Advances in developmental neurosciences have strengthened this concept by demonstrating that schizophrenia is characterized by abnormalities in spine maturation, neuronal connectivity, and cortical circuit development [21]. Reduced dendritic spine density, impaired synaptic plasticity, and excessive synaptic pruning have emerged as consistent neuropathological features of schizophrenia, suggesting that disruptions in mechanisms governing neuronal maturation begin long before disease onset [21]. These developmental processes are regulated by intricate signalling networks that extend beyond classic neurodevelopmental pathways. Increasing evidence suggests that immune signalling pathways interact with developmental regulators, particularly Wnt5–β-catenin signalling pathways. Therefore, a balance between cytokines and developmental signalling pathways appears to be critical for establishing neuronal architecture, maintaining synaptic integrity, and supporting appropriate neural circuit formation.

Several cytokines have been identified as key modulators of neurodevelopment, including interleukins (IL-1β, IL-6, IL-8, and IL-33), tumour necrosis factor-alpha (TNF-α), and interferon-gamma (IFN-γ) [22]. During neurodevelopment, these cytokines tightly regulate feedback networks that coordinate communication between neurons, astrocytes, microglia, and other glial cells [23,24]. This is often characterized by astrocytes secreting substantial amounts of IL-33 during neurodevelopment [25]. IL-33 is increasingly recognised as a key regulator of neural communication. IL-33 promotes microglial survival, enhances phagocytic capacity, and supports cellular metabolism through the activation of the IL-33/ST2/AKT signalling pathway [26]. This signalling cascade facilitates synaptic remodelling and supports optimal neuronal synapse formation. High concentrations of IL-33 are found in the thalamus and cerebellar hemispheres, which support further regional neuronal maturation by enhancing microglial capacity to clear cellular debris, apoptotic cells, and toxic proteins [27]. Through these actions, IL-33 protects developing neurons from neurotoxicity and fosters an environment conducive to optimal neuronal maturation [27].

IL-1β, despite being a pro-inflammatory cytokine, also performs essential physiological functions during neurodevelopment. Experimental studies have demonstrated that physiological levels of IL-1β promote neuronal proliferation, differentiation, migration, and apoptosis in the developing brain [28]. These effects appear to be mediated through close interactions with Wnt5a signalling, which regulates cellular proliferation, polarity, migration, adhesion, and neuronal differentiation [28]. Notably, IL-1β and Wnt5a appear to regulate each other in bidirectional relationships during neurogenesis [28]. In addition, IL-1β modulates the expression of neurotrophin-3 (NT-3), an essential neurotrophic factor involved in neuronal proliferation, differentiation, and survival throughout life [29]. Similarly, experimental studies have shown that IFN-γ promotes neuronal development by activating the Wnt7a–β-catenin and Cyclin D1 signalling pathways [30,31]. However, dysregulated cytokine milieu, particularly elevated levels of pro-inflammatory cytokines during neurodevelopment, may disrupt normal brain maturation and increase vulnerability to schizophrenia and other primary psychotic disorders [22].

## 4. Mechanisms Behind Immune Dysregulation in Schizophrenia

Growing evidence suggests that both genetic susceptibility and environmental exposures contribute to the development of inflammatory abnormalities. Among the environmental factors, maternal immune activation during pregnancy and childhood trauma have emerged as among the consistently implicated risk factors. The role of substances, while notable, is less straightforward. Although substances can induce significant alterations in inflammatory markers, the neurobiological mechanisms underlying substance-induced psychotic disorders differ substantially from those involved in primary psychotic disorders such as schizophrenia. In addition, substance-induced psychosis often follows a distinct clinical course, with inflammatory changes influenced by the type, duration, and severity of substance exposure.

### 4.1. Maternal Infections and Risk of Inflammation in Offspring

Evidence suggests that there is a strong link between elevated cytokines during pregnancy and risk of psychosis in offspring [22]. Early work by Buka et al. (2001) highlighted a strong association between increased maternal TNF-α levels and the risk of schizophrenia in adult offspring, with mothers of affected individuals exhibiting significantly higher concentrations of this cytokine compared to mothers of unaffected adults [32]. Supporting this, Allsewede et al. (2020) conducted a longitudinal study and found that mothers whose offspring later developed schizophrenia had significantly higher serum levels of TNF-α, IL-1β, and IL-6 compared to mothers of non-affected offspring [33]. However, the literature is not entirely consistent. For example, Buka et al. (2001) did not find significant differences in IL-1β and IL-6 levels between cases and controls [32], while Brown et al. (2004) reported that only IL-8 levels were elevated in mothers of affected offspring, with no differences observed in other cytokines (IL-1β, IL-6, or TNF-α) [18]. These findings suggest that while maternal immune activation is a risk factor for psychosis in offspring, cytokine patterns vary, reflecting complex immune responses during pregnancy. More research is needed to determine which aspects of maternal inflammation most influence psychiatric disorders in children.

### 4.2. Childhood Trauma Exposure and Elevated Cytokines

Childhood trauma exposure (CTE) has been shown to be linked to increased immune activation, particularly through the upregulation of the pro-inflammatory cytokines [34]. Early-life stress such as CTE can result in sustained elevations in cortisol, a glucocorticoid hormone that, despite its immunoregulatory role, can promote chronic low-grade inflammation [35]. Studies have also shown that individuals exposed to CTE, whether healthy or diagnosed with schizophrenia, display a pro-inflammatory immune profile characterized by elevated levels of cytokines, including IL-6, IL-1β, TNF-α, and IFN-γ [36,37,38]. For example, Hartwell et al. (2013) and Quide et al. (2019) found significant associations between CTE and elevated proinflammatory cytokine levels in both healthy adults and individuals with schizophrenia [36,38]. Trauma-exposed groups consistently exhibited higher concentrations of IL-6 and TNF-α than their non-exposed counterparts [36,38]. Prior to this, Dennison et al. (2012) had reported that trauma-exposed individuals with schizophrenia had higher cytokine levels than schizophrenia patients and healthy controls without trauma exposure [39]. Recently, Yi et al. (2022) reported increased plasma IL-6 and TNF-α in adults with a history of CTE, alongside a higher risk of developing schizophrenia [40]. However, some studies, such as Allison and Upthegrove (2020) and Counotte et al. (2019), did not find a significant difference in cytokine concentrations or a clear association with risk of psychosis among trauma-exposed individuals [41,42].

## 5. Cytokine and Neurotransmission in Psychosis

Converging evidence implicates that dopamine and glutamate dysregulation in primary psychosis may be accompanied by immune dysregulation. Disruptions in these neurotransmitters are considered central in the manifestation of cognitive, emotional, and perceptual symptoms of psychotic disorders [43]. Excessive dopamine activity is linked to positive symptoms through aberrant salience attribution, while deficits in prefrontal dopamine contribute to negative symptoms and cognitive impairment. Glutamate dysfunction, particularly via NMDA receptor hypofunction, has been shown to induce psychosis-like states in experimental settings [44]. Importantly, mounting evidence demonstrates that inflammatory processes bridge these neurotransmitter systems [45]. Cytokines can modulate dopamine signalling and are elevated in states of increased dopaminergic activity, such as cocaine use, thereby enhancing the production of pro-inflammatory mediators, including IL-1β, TNF-α, IL-2, IL-6, and IL-17 [46,47,48]. Dopamine also acts as an immune regulator, with elevated levels promoting neuroinflammation and increasing IL-1β via NF-κB and the NLRP3 inflammasome [47]. For individuals who use cocaine, a pronounced surge in dopamine neurotransmission has been reported. In cocaine users, elevated synaptic dopamine correlates with increased IL-1β levels [48]. Similarly, correlations between peripheral inflammatory cytokines and central glutamate levels, including associations between IFN-γ or IL-8 and caudate glutamate in FEP and treatment-resistant patients, suggest that immune dysregulation and neurotransmitter imbalance are tightly interconnected in primary psychosis [49].

## 6. Cytokines in the First Episode of Psychosis

A pro-inflammatory cytokine profile has been observed in FEP, with studies frequently reporting elevated levels of cytokines such as IL-6, IL-10, TNF-α, and others [50]. However, these findings are not consistent across all studies, likely due to both methodological and biological differences [51]. Some studies have demonstrated increased levels of TNF-α, IFN-γ, and TNF-β, along with reduced anti-inflammatory cytokines, in FEP patients compared to controls, whereas others have reported elevated levels of IL-1α, IL-1β, and IL-8 [34,52,53]. The evidence for individual cytokines remains mixed: for example, Di Nicola et al. (2013) and Kubistova et al. (2012) reported higher IL-6 levels in FEP, whereas Mishra et al. (2026) and Petrikis et al. (2015) reported lower levels [34,53,54,55]. Similarly, findings about IFN-γ and IL-2 are inconclusive, as some studies note increases in psychosis, but results are confounded by mixed samples including both FEP and chronic schizophrenia [56]. The expression of anti-inflammatory cytokines, such as IL-2 and IL-10, is also unclear, with some studies reporting increases and others finding no significant differences between patients and controls [34,55]. Overall, there is evidence to suggest that a pro-inflammatory state may contribute to the onset of psychosis.

## 7. Cytokines and Disease Severity and Outcomes in the First Episode of Psychosis

Recent research examining the relationship between cytokines and symptom domains in psychosis reveals both converging and divergent findings, with several studies highlighting specific associations for IL-6, TNF-α, and other cytokines. Multiple investigations have reported that elevated IL-6 levels are associated with impaired cognitive performance in psychosis, particularly slower processing speed, deficit syndrome, and negative correlations with information processing [57,58,59]. However, these associations are not universally observed, as evidenced by Qwabe et al. (2026), who reported no significant link between IL-6 and total or subscale PANSS scores [51].

Similar variability is evident for TNF-α. Higher TNF-α levels have been linked to impaired facial and emotional recognition and increased depression and agitation symptoms, as well as correlations with PANSS total, positive, general psychopathology, and negative symptom domains, including blunted affect and alogia [57,59,60,61]. Conversely, findings for other cytokines are less consistent or less well-established. For instance, while elevated IL-4 levels have been associated with higher PANSS depression scores, no significant relationship with overall PANSS scores has been found [60]. Additionally, IL-8 and IL-10 have shown no significant associations with PANSS scores or subscales [48]. However, Xu et al. (2025) reported elevated IL-8 levels in individuals experiencing acute psychosis, demonstrating a significant association with the PANSS anxiety/depression subscales [62]. Overall, while certain cytokines such as IL-6 and TNF-α exhibit notable associations with specific symptom domains in psychosis, inconsistencies across studies highlight the need for further research to clarify these relationships.

## 8. Effects of Antipsychotics on Cytokines in FEP

Antipsychotics are increasingly recognized for their effects on immune function in individuals with psychosis. Recent research demonstrates that these medications modulate cytokine profiles, reflecting a complex interaction between antipsychotic therapy and immunological processes. Given the association between a pro-inflammatory state and psychosis severity, it is plausible that effective antipsychotic therapy may reduce pro-inflammatory cytokines while increasing anti-inflammatory cytokines. Several studies have reported little or no significant change in selected inflammatory markers despite clinical improvement, highlighting the heterogeneity of immune responses in FEP.

Clinical studies, including Borovcanin et al. (2013), Petrikis et al. (2015), and Kalyoncu et al. (2025), demonstrated that antipsychotic treatment in patients with FEP who exhibited elevated pre-treatment IL-6 levels (compared to healthy controls) resulted in a significant reduction in IL-6 following treatment, indicating anti-inflammatory effects [55,63,64]. The anti-inflammatory effects of antipsychotic treatment correlate positively with improvements in symptoms. Similarly, significant post-treatment reductions in IL-1β and IL-27 were reported by Kalyoncu et al. (2025) and Borovcanin et al. (2013), respectively [63,64]. In contrast, TNF-α, although elevated at baseline, did not demonstrate a significant reduction but rather showed a trend towards normalization following treatment in the study by Noto et al. (2015) [65]. Among the anti-inflammatory cytokines, Noto et al. (2015) reported that IL-10 was elevated at baseline and generally trended towards normalization following treatment, while both Noto et al. (2015) and Borovcanin et al. (2013) observed reductions in IL-4 after antipsychotic therapy [63,65].

## 9. Adjuvant Anti-Inflammatory Mediators and Treatment Response in Psychosis

Although definitive evidence establishing inflammation as a therapeutic target in primary psychosis is still emerging, growing recognition of immune dysregulation has shifted attention towards immunomodulatory strategies as potential adjuncts to conventional antipsychotic treatment. This evolving paradigm has prompted investigations into both pharmacological and non-pharmacological interventions to attenuate inflammation and improve clinical outcomes in individuals with FEP.

Several adjunctive pharmacotherapies have demonstrated promising, albeit variable, results. Zarghami et al. (2024) reported that adjunctive celecoxib with haloperidol significantly improved total PANSS scores, positive symptoms, and general psychopathology over a five-week period compared to haloperidol alone, although cytokines were not measured, limiting conclusions regarding the underlying mechanism [66].

Zhang et al. (2018) evaluated the relationship between symptom improvement and inflammatory markers in a placebo-controlled trial of adjunctive minocycline in patients receiving risperidone [67]. Participants receiving minocycline experienced a significant reduction in negative symptoms compared with those receiving a placebo, and this improvement strongly correlated with a reduction in IL-1β.

Weickert et al. (2024) investigated adjunctive canakinumab and found no significant between-group differences in IL-6, IL-1β, or IL-8 concentrations following treatment [68]. Nevertheless, both treatment groups demonstrated significant reductions in IL-6 and IL-1β over time, with a trend towards greater cytokine reductions in the canakinumab group. Positive symptoms also improved significantly, although no additional benefits were observed for negative symptoms or cognitive performance [68].

Non-pharmacological interventions have likewise shown potential immunomodulatory effects. Dunleavy et al. (2024) demonstrated that a six-week programme of moderate-to-vigorous exercise significantly reduced circulating IL-6 concentrations, although no significant changes were observed in IL-8 or IL-10 levels [69]. Exercise was also associated with significant improvements in negative symptoms, whereas positive and cognitive symptoms remained largely unchanged [69].

These findings suggest that immunomodulatory interventions, whether pharmacological or behavioural, may complement conventional antipsychotic therapy by reducing inflammatory activity and improving clinical outcomes, particularly for negative symptoms. However, the heterogeneity of cytokine responses and variable clinical efficacy across studies underscore the need for larger, well-designed longitudinal trials to identify the patients most likely to benefit from targeted immunomodulatory therapies and to clarify the mechanisms linking immune modulation to symptom improvement.

## 10. Conclusions

This narrative review highlights the growing body of evidence supporting a role for immune dysregulation in the pathogenesis and clinical expression of FEP. Epidemiological, experimental, and clinical studies collectively suggest that maternal immune activation during pregnancy and childhood trauma are important environmental risk factors that may increase vulnerability to psychosis through alterations in inflammatory pathways [70,71]. Although cytokines are essential regulators of normal neurodevelopment, dysregulation of these immune mediators, particularly pro-inflammatory cytokines such as IL-6, TNF-α, and IL-1β, may contribute to aberrant neurodevelopment, neurotransmitter dysfunction, and the emergence of psychotic symptoms.

The available literature demonstrates a pro-inflammatory state in a subgroup of individuals with FEP; however, the expression of individual cytokines remains heterogeneous across studies. Differences in study populations, methodological approaches, cytokine assays, and clinical characteristics likely contribute to these inconsistencies. Nevertheless, accumulating evidence suggests that specific cytokines, particularly IL-6 and TNF-α, may be associated with cognitive impairment, negative symptoms, depression, and disease severity, indicating that immune abnormalities may contribute not only to the onset but also to the clinical heterogeneity of psychosis.

Conventional antipsychotic treatment appears to have partial immunomodulatory effects, with reductions in several pro-inflammatory cytokines, most consistently IL-6 and IL-1β. Emerging evidence further suggests that adjunctive immunomodulatory interventions, including anti-inflammatory pharmacotherapies and behavioural interventions such as exercise, may confer additional clinical benefits, particularly for persistent negative symptoms. However, findings remain inconsistent, and current evidence is insufficient to support the routine implementation of immune-targeted therapies in clinical practice. Overall, the evidence supports the concept that immune dysregulation is an important component of the pathophysiology of psychosis rather than a universal mechanism applicable to all patients.

## 11. Strengths and Limitations

This review provides an integrated narrative synthesis of current evidence linking immune dysregulation to the onset and trajectory of FEP. By synthesising epidemiological, neurodevelopmental, immunological, and clinical perspectives, it presents a biological framework that enhances our understanding of the disorder. The discussion covers potential pharmacological and non-pharmacological immunomodulatory interventions, with particular emphasis on FEP. Furthermore, the review explores the interplay between cytokine imbalances and neurotransmitter disruptions and their links to clinical symptoms, offering insight into the disorder’s underlying mechanisms. The review has notable limitations. Its narrative approach is vulnerable to selection bias and lacks the methodological rigour typically associated with systematic reviews. The absence of a formalized systematic search strategy, such as PRISMA, restricts transparency and reproducibility.

## 12. Future Studies

Future research should prioritize addressing both methodological and biological heterogeneity that currently hinders interpretation in this field. To enable more reliable comparisons across studies, it is essential to standardize cytokine measurement protocols, sampling procedures, and analytical techniques. Large, prospective longitudinal studies are needed to map immune alterations from the prodromal stage through chronic psychosis, helping clarify disease trajectories and critical periods for intervention. Incorporating maternal tissues such as placenta and umbilical cord blood into future study designs is also vital, as these sources can provide unique insights into prenatal immune exposures and their role in neurodevelopment. Considering epigenetic mechanisms in these tissues will further elucidate how early-life environmental factors influence gene expression and long-term vulnerability to psychosis. Identifying robust peripheral and central inflammatory biomarkers is also crucial for predicting disease onset, monitoring symptom progression, guiding treatment selection, and informing prognosis. A deeper mechanistic understanding can be achieved by integrating multimodal methods such as genomics, transcriptomics, proteomics, neuroimaging, and metabolomics to explore how cytokine dysregulation interacts with neurodevelopment, neurotransmitter systems, and neural circuitry. Furthermore, evaluating adjunctive immunomodulatory interventions, including pharmacological agents and behavioural strategies such as exercise, using adequately powered randomized controlled trials may offer new avenues for treating negative symptoms and cognitive dysfunction. These research directions will deepen our understanding of the immunopathogenesis of psychosis and pave the way for personalized therapeutic strategies that integrate immune biomarkers with conventional clinical assessment.

## Data Availability

No new data were created or analysed in this study.
